# Genome-wide screening of upstream transcription factors using an expression library

**DOI:** 10.12688/f1000research.27532.2

**Published:** 2021-03-08

**Authors:** Naoya Yahagi, Yoshinori Takeuchi

**Affiliations:** 1 Nutrigenomics Research Group, Faculty of Medicine, University of Tsukuba, Tsukuba, Ibaraki, 3058575, Japan

**Keywords:** Transcription, Transcription factor, Expression cloning

## Abstract

The identification of upstream transcription factors regulating the expression of a gene is generally not an easy process.  To facilitate this task, we constructed an expression cDNA library named Transcription Factor Expression Library (TFEL), which is composed of nearly all the transcription factors in the mouse genome. Genome-wide screening using this library (TFEL scan method) enables us to easily identify transcription factors controlling any given promoter or enhancer of interest in a chromosomal context-dependent manner. Thus, TFEL scan method is a powerful approach to explore transcriptional regulatory networks.

## Abbreviations

TFEL, transcription factor expression library; VEGF, vascular endothelial growth factor; Fasn, fatty acid synthase; SREBP, sterol regulatory element-binding protein; HIF, hypoxia-inducible factor; LXR, liver X receptor; KLF, Kruppel-like factor.

## Introduction

Transcriptional regulation is widely involved in many biological processes of relevance such as cellular development, tissue differentiation, reprogramming and apoptosis as well as nutrient metabolism; in many cases, such processes involve complicated regulatory networks ^1^. Generally, downstream targets of a transcription factor can be identified in a convincing way through transcriptome analyses after overexpression or knockdown of the transcription factor, or through investigating the binding interaction between the transcription factor and genomic DNA using chromatin immunoprecipitation (ChIP)-sequencing method. However, no assured methods of identifying upstream transcription factors regulating a specific promoter or enhancer exists, since the relatively poor amount of transcription factors expressed in protein pools or cDNA libraries makes purification difficult, while binding factor prediction using computer analysis based on binding motif databases suffers from high false-positive and false-negative rates in general ^2^. Therefore, we constructed a comprehensive expression library of transcription factors in the mouse genome, the Transcription Factor Expression Library (TFEL), and developed a new method of identifying upstream transcription factors through an expression cloning technique using the TFEL (TFEL scan method). Using this TFEL scan method, we can easily identify the specific transcription factor or transcription factor complex trans-acting against any given cis-element of interest.

## Methods

### Retrieving clones from DNABook

The mouse transcription factor DNABook™ was purchased from DNAFORM, Tokyo, Japan ^3^. Each spot on the sheets of DNABook was punched, and plasmid DNA was eluted in 10μl distilled water. Competent DH5α E. coli was transformed with 1μl of the eluted DNA solution, and plasmids were cloned in a standard manner.

 Among 1,588 clones in the DNABook, 1,350 clones were on pFLCI vector, 204 clones on modified BluescriptI(+), 23 clones on pFLCIII, and 11 clones were on pFLCII.

The clones on pFLCI were digested by restriction enzymes EcoRI and BamHI, clones on pFLCII by XhoI and BamHI, pFLCIII by Bsu36I and BamHI, and clones on modified BluescriptI(+) were cut by SacI and XhoI on a 37 °C heat block. The restricted DNA fragments were separated on agarose gel (Cat. # 5091, Agarose Basic TAKARA) by electrophoresis, and verified if they are correspondent with the predicted band pattern calculated from the database (GenBank / EMBL / DDBJ) sequence. Unfortunately, information about which clones are included in the DNABook cannot be made public due to a confidentiality agreement. For more details, please contact the corresponding author via email ( nyahagi-tky@umin.ac.jp)

### Construction of TFEL

TFEL was constructed by transferring all the DNABook clones into pcDNA3.1 expression vector (Invitrogen). To facilitate this process, we first modified pcDNA3.1 vector and inserted the PCR-amplified kanamycin-resistant gene from pENTR4 vector (Invitrogen) into the original pcDNA3.1 at the SmaI-SalI sites by replacement of neomycin-resistant gene to make the vector resistant to kanamycin. By this modification, we could ligate an insert DNA fragment to the pcDNA3.1 vector directly without agarose gel isolation of the insert from vector backbone fragment and undigested plasmid after restriction enzyme digestion of DNABook clones, which are ampicillin-resistant.

Because DNABook clones were on as many as 23 versions of various vectors (pFLCI: 12, pFLCII: 3, pFLCIII: 6, and modified BluescriptI(+): 2), multiple strategies based on the calculation of restriction sites were used to construct TFEL clones.

For pFLCI clones, the combination of EcoRI and BamHI digestion was first tried to cut insert fragments out of vectors when inserts were not predicted to be cut by the two restriction enzymes, and excised fragments were inserted into pcDNA3.1(-) vector at EcoRI and BamHI sites. For pFLCI clones whose inserts were cut by EcoRI or BamHI, SfiI digestion and ligation to pcDNA3.1(-)-SfiI (XhoI-EcoRI part of pcDNA3.1(-) multiple cloning site was replaced with synthesized SfiI sequence) was next attempted. For clones to which neither strategy was applicable, either EcoRI-ApaI (into pcDNA3.1(+)), EcoRI-KpnI (into pcDNA3.1(-)), or SacI-BamHI (into pcDNA3.1(+)-SacI-mod whose SacI site is modified to be unique by introducing mutation to the other SacI site outside of the multiple cloning site) strategy was tried. For the other 73 clones to which these strategies were not applicable, SfiI digested multiple fragments were inserted into pcDNA3.1(-)-SfiI at one time. When another restriction enzyme such as ApaI, KpnI, NotI, or ScaI could be used to cut the vector backbone without cutting the insert, this treatment was done at the same time to reduce the possibility of a vector fragment being cloned into pcDNA3.1.

For modified BluescriptI(+) clones, the SfiI (into pcDNA3.1(-)-SfiI) or XhoI-SacI (into pcDNA3.1(-)-SacI-mod) strategy was used. For 47 plasmids on ZA vector whose orientation of the insert is opposite to that of others, corresponding “(+)” version of pcDNA3.1 vectors were used. 7 clones on ZX vector had corrupted 5’-side SfiI and XhoI sites, therefore KpnI-SfiI, KpnI-BglII, or BssHII strategy was used instead.

For pFLCII clones, the XhoI-BamHI strategy was used when applicable. Others were excised with SacI and cloned into SacI site of pcDNA3.1(-)-SacI-mod, after which the inserted direction was checked, and clones constructed in the correct direction was selected.

pFLCIII clones were ligated using I-CeuI and PI-SceI.

### Functional screening through luciferase activity

The mouse *Vegfa*-luc plasmid (-1210 to +246) was generated from a PCR-amplified fragment inserted into pGL2-basic vector (Cat. #E1641, Promega) at SmaI site. The PCR primers used were 5’-AAGATGAACCGTAAGCCTAGGCT-3’ and 5’-AACCGTTGGCACGATTTAAGA-3’ and amplification was performed according to a 3-step method. The rat *Fasn*-luc plasmid (-397 to +28) was prepared as described previously ^4^. The mouse *Srebf1c*-luc plasmids (-2.2k to +40 and -249 to -144) were constructed as described elsewhere ^5, 6^.

To screen transcription factors regulating a target promoter, TFEL clones were co-transfected with the specific promoter-luc plasmid into HEK293 cells using SuperFect Transfection Reagent (Cat. #301305, QIAGEN). The screenings were performed by co-transfecting 10–20 clones with each luciferase plasmid per one well. Transfection was performed using 0.5μg of plasmid DNA pooled equally from 10 or 20 TFEL clones per well. The luciferase activity in transfectants was measured on a luminometer (BERTHOLD) with a standard assay kit (Cat. #E1483, Promega). First screen was performed with 10 or 20-clone pools per well, and significantly shifted pools were further tested with one clone at a time.

## Data analysis

All the data from a luminometer were output as Excel sheets, and directly input to graphs. All the graphs were drawn in Microsoft Excel 2016 (16.0.5083.1000).

## Results

### Identification of binding transcription factor

**Figure 1.**
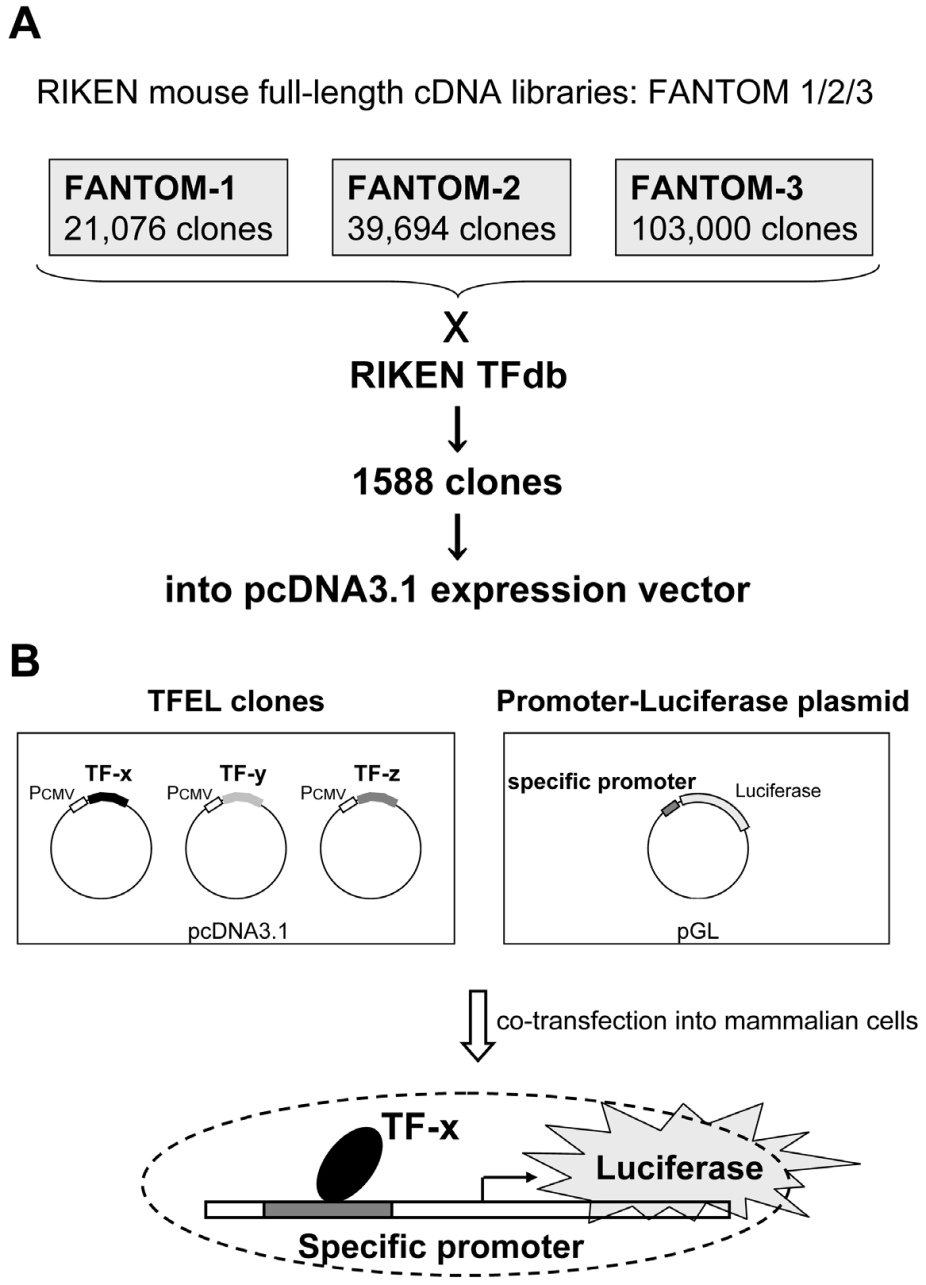
( **A**) Construction of Transcription Factor Expression Library (TFEL). The original clones were derived from the RIKEN FANTOM1/2/3 libraries, and 1,588 clones that are predicted to be transcription factor genes by RIKEN TFdb are distributed as Mouse Transcription factor DNABook™ from DNAFORM Inc. TFEL consists of the DNABook clones transferred into pcDNA3.1 expression vectors through recombination using several restriction enzymes optimized to each clone. ( **B**) Workflow of identifying upstream transcription factors using TFEL. A luciferase plasmid with a specific promoter of interest is co-transfected with the TFEL clone(s) into mammalian cells and the luciferase reporter activities are measured.

To construct the TFEL expression plasmid library, we used clones from the RIKEN FANTOM libraries ( Figure 1A). The clone subset consisting of genome-wide transcription factors is available from DNAFORM as Mouse Transcription factor DNABook™, and is comprised of 1,588 nonredundant genome-wide mouse transcription factor genes ^7^. We retrieved all the clones in the DNABook using *Escherichia coli* transformation. After checking the band patterns using several restriction enzymes and sequencing if necessary, each excised insert DNA fragment was transferred to a pcDNA3.1 expression vector. In this manner, we constructed a pcDNA3.1 expression library composed of 1,588 clones of genome-wide and nonredundant mouse transcription factors.

**Figure 2.**
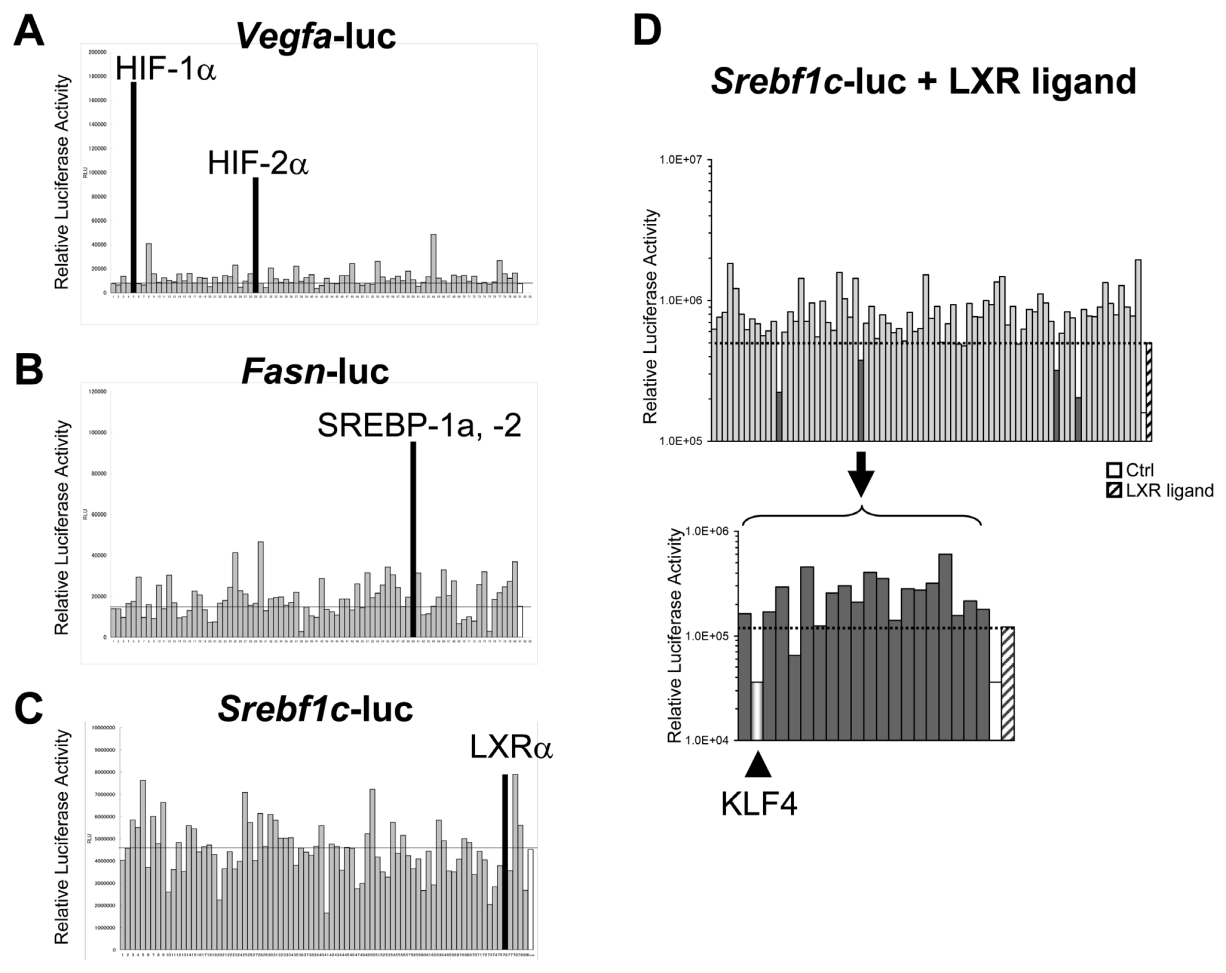
( **A**– **C**) Representative cases of the analysis of upstream transcription factors using Transcription Factor Expression Library (TFEL) against specific promoters: ( **A**) vascular endothelial growth factor (gene name: *Vegfa*), ( **B**) fatty acid synthase (gene name: *Fasn*), and ( **C**) sterol regulatory element-binding protein-1c (gene name: *Srebf1c*). Transfection was performed using 0.5μg of plasmid DNA pooled equally from 10 or 20 TFEL clones per well. ( **D**) Representative case identifying the combinatorial interactions on a specific promoter. In this assay, we screened for transcription factor(s) that suppress LXR activity on the SREBP-1c promoter (-249 to -144), because we had found an important element shown to exert a suppressive effect on the SREBP-1c promoter in a fasting state ^6^. To activate LXR and to improve the sensitivity of the assay, we added an LXR ligand T0901317 to the culture media. The first screening was performed using a 20-clone pool assay, and individual clones were examined in the second screening. From the statistical point of view, we picked up the top (or the bottom for D) 5% pools for the 2nd screening, and as a result, this criterion was similar to the 2SD (standard deviation) criterion in these cases.

Next, we evaluated the validity of this expression cloning method (TFEL scan method) using the TFEL ( Figure 1B ^8^). Figure 2A–C ^8^ shows three cases in which the upstream transcription factors influencing well -known promoters were examined: A, vascular endothelial growth factor (gene name: *Vegfa*), B, fatty acid synthase (gene name: *Fasn*), and C, sterol regulatory element-binding protein (SREBP)-1c (gene name: *Srebf1c*). In these cases, well-known determinant transcription factors (HIF-1α and -2α for *Vegfa*-luc, SREBP-1a and -2 for *Fasn*-luc, and LXRα for *Srebf1c*-luc) were identified as expected. These screenings were performed by co-transfecting 10–20 clones with each luciferase plasmid per one well. 

These results demonstrated that the identification of upstream transcription factors using this expression screening method works well.

### Identification of transcription factor complex

We further attempted to identify unknown partner(s) interacting with a specific transcription factor on a specific gene promoter. To this end, we screened interacting partner(s) for LXR on the SREBP-1c promoter ^6^. As shown in Figure 2D ^9^, KLF4 was picked up as a candidate from the first screening performed by co-transfecting 20 clones with an SREBP-1c promoter luciferase plasmid in the presence of an LXR ligand T0901317. Further screening of the clones one by one revealed that KLF4 and KLF15 interacted with and suppressed LXR on the SREBP-1c promoter (data not shown, see *ref* 6). Thus, the TFEL scan method was proved to be useful for searching the interacting partner against a specific transcription factor of interest in a DNA-sequence-dependent manner.

## Discussion

In the present study, we clearly demonstrated that our new method of TFEL scan is a powerful approach to explore transcriptional regulatory networks by identifying transcription factor(s) involved in the regulation.

First, we showed that in three cases ( *Vegfa*-luc, *Fasn*-luc and *Srebf1c*-luc), we could easily identify well-known determinant transcription factors (HIF-1α and -2α for *Vegfa*-luc, SREBP-1a and -2 for *Fasn*-luc, and LXRα for *Srebf1c*-luc) as expected ( Figure 2A–C ^8^). In addition, we recently succeeded in identifying the binding transcription factor for an important SNP at the diabetes-associated *TCF7L2* gene locus through our TFEL scan method ^10^. It is suggested that *TCF7L2* is the single largest effect of a common SNP on type 2 diabetes risk in European populations ^11^.

Thus, we demonstrated that the TFEL scan method works very well to identify the regulatory transcription factor in a simple model. It is noteworthy that the TFEL scan method is also applicable to enhancers as well as promoters as shown in the case of *TCF7L2* gene.

Many of the transcription factors characterized thus far are responsive to stresses or ligands. The results of the case of LXR shown here demonstrate that such transcription factors can also be identified by overexpression. However, at the same time, we have experienced that some transcription factors activated in a ligand-dependent manner, for example, glucocorticoid receptor (GR), cannot be identified by mere overexpression without ligands (data not shown). Therefore, it may depend on transcription factors whether they can be detected without ligands/additional activators or not.

Next, we additionally succeeded in identifying transcription factors forming a complex such as LXR and KLF4 ( Figure 2D ^8^). Therefore, the TFEL scan method was proved to be similarly effective even in these more complex cases.

It is well known that the transcriptional output of a gene is due to the joint activity of many transcription factors, the binding and activation of which are highly interdependent ^1^. This cooperation is often mediated by direct physical contact between two or more transcription factors, forming homodimers, heterodimers, or larger transcriptional complexes ^12^. In fact, it has been estimated that approximately 75% of all metazoan transcription factors heterodimerize with other factors ^13^. Thus, the importance of transcription factor combinations has been highlighted more and more, and the mapping of the combinatorial interactions among transcription factors has been attempted using a mammalian two-hybrid system ^12^. Our successful results identifying KLFs as interacting partners of LXR on *Srebf1c* promoter clearly showed that the expression cloning method using the TFEL (TFEL scan method) may also be useful for exploring interacting partner(s) among transcription factors on a specific DNA fragment of interest. In contrast, binding site prediction based on binding motif databases suffers from high false-positive and false-negative rates in general ^9^. In particular, the sequence-based approach has no power to predict transcription factor complexes formed by protein-protein interaction. Conversely, the search for protein-protein interactions among transcription factors alone, for example, using a mammalian two-hybrid system, may not be sufficient to elucidate the regulatory complexes in certain situations including the present case of LXR-KLFs complex, because complexes like LXR-KLFs are also dependent on DNA binding and therefore locus-specific. This relatively weak interaction supported by the DNA backbone enables gene-specific regulations and may possibly give more diversity to transcriptional networks. Thus, our strategy of screening for the transcription factor complex in a chromosomal context-dependent manner using the TFEL scan method can be a very effective and powerful approach for exploring sophisticated transcriptional networks in detail, because this screening system is performed under more physiological conditions than traditional methods.

Our initial approach using a mixed pool assay handling 20 clones at a time failed to detect KLF15 in the first screening described above. Interference among the co-transfected transcription factors in the pools was thought to have affected the results and veiled the KLF15-LXR interaction, suggesting that a single clone assay is preferable, especially for screening for combinatorial interactions. In relation to this point, interactions with endogenous transcription factors should also affect the results to varying degrees, and screenings in different cell lines should provide more information.

Our TFEL library is based on the Mouse Transcription factor DNABook™, and a small portion of transcription factors are missing, as they previously reported ^7^. For example, among the 17 KLF family members, 4 KLFs (KLF7, 12, 14 and 17) are missing. We are planning to fix this problem and to complete the construction of a comprehensive expression library in the near future.

In summary, the present study clearly demonstrates that our expression cloning method using the TFEL (TFEL scan method) enables us to efficiently identify regulatory transcription factors as well as to elucidate combinatorial interactions among transcription factors in a chromosomal context-dependent manner. Thus, the TFEL scan method is a powerful approach to explore transcriptional regulatory networks.

## Data availability

### Underlying data

Figshare: Underlying data of Figure 2. Representative cases of the analysis of upstream transcription factors using TFEL against specific promoters. https://doi.org/10.6084/m9.figshare.13237319 ^8^

This project contains the following underlying data:

- Fig2A_090109_BxVEGF1.3k.xls

Representative cases of the analysis of upstream transcription factors using TFEL against specific promoters: (A) vascular endothelial growth factor (gene name: Vegfa).

- Fig2B_081218_AxFAS0.4k.xls

Representative cases of the analysis of upstream transcription factors using TFEL against specific promoters: (B) fatty acid synthase (gene name: Fasn).

- Fig2C_101026_B(10)xSREBP1c2.2k.xls

Representative cases of the analysis of upstream transcription factors using TFEL against specific promoters: (C) sterol regulatory element-binding protein-1c (gene name: Srebf1c).

- Fig2D_081219_AxSREBP1c-T_1st.xls

Representative case identifying the combinatorial interactions on a specific promoter. In this assay, we screened for transcription factor(s) that suppress LXR activity on the SREBP-1c promoter (-249 to -144). 1st screening data.

- Fig2D_081225_SREBP1c-T_2nd_A28.xls

Representative case identifying the combinatorial interactions on a specific promoter. In this assay, we screened for transcription factor(s) that suppress LXR activity on the SREBP-1c promoter (-249 to -144). 2nd screening data.

Data are available under the terms of the Creative Commons Attribution 4.0 International license (CC-BY 4.0).
