# Supplementary material for: Genome-wide screening of upstream transcription factors using an expression library
Source: F1000Res. 2021 Mar 8;10:51. Originally published 2021 Jan 28. [Version 2] doi: 10.12688/f1000research.27532.2 (PMC7968528; doi:10.12688/f1000research.27532.2)
Supplement: Peer review history

# Peer review: Reviewer response for version 2

**DOI:** 10.5256/f1000research.55150.r81021

**Published:** 2021-03-16

**Authors:** Harukazu Suzuki

**Affiliations:** 1 Laboratory for Cellular Function Conversion Technology, RIKEN Center for Integrative Medical Sciences, Yokohama, Japan

I have no additional comments.

Is the work clearly and accurately presented and does it cite the current literature?

Yes

If applicable, is the statistical analysis and its interpretation appropriate?

No

Are all the source data underlying the results available to ensure full reproducibility?

Yes

Is the study design appropriate and is the work technically sound?

Yes

Are the conclusions drawn adequately supported by the results?

Yes

Are sufficient details of methods and analysis provided to allow replication by others?

Yes

Reviewer Expertise:

Regulation of gene expression, TF-mediated DNA demethylation, Cell reprogramming

I confirm that I have read this submission and believe that I have an appropriate level of expertise to confirm that it is of an acceptable scientific standard.

# Peer review: Reviewer response for version 1

**DOI:** 10.5256/f1000research.30430.r79133

**Published:** 2021-02-19

**Authors:** Harukazu Suzuki

**Affiliations:** 1 Laboratory for Cellular Function Conversion Technology, RIKEN Center for Integrative Medical Sciences, Yokohama, Japan

This paper describes a method to identify transcription factors (TFs) regulating target gene(s). The expression constructs that authors has made are highly useful for scientific community who are interested in TF-mediated gene regulation. The manuscript is written well, but there are several issues to be addressed.

1. Identification of upstream TF(s) should be objective. Test cases shown in Figure 2A, B and C should be statistically evaluated. HIF-1a,2a and SREBP-1a,-2 may be evident, however I am not sure whether LXRa-mediated luciferase activity is significantly high or not.
2. In figure 2D, authors selected 4 pools. I think this selection should also be objective by using statistical evaluation. If authors statistically selected pools for further one by one test, the pool with KLF4 has been selected?
3. One of the most important value in this work must be TF expression plasmids that authors have systematically constructed. I think this resource should be available to scientific community.
4. TFs regulate downstream genes by associating with promoters or enhancers. It is worth if the screen system works for TFs associating with enhancers.

Is the work clearly and accurately presented and does it cite the current literature?

Yes

If applicable, is the statistical analysis and its interpretation appropriate?

No

Are all the source data underlying the results available to ensure full reproducibility?

Yes

Is the study design appropriate and is the work technically sound?

Yes

Are the conclusions drawn adequately supported by the results?

Yes

Are sufficient details of methods and analysis provided to allow replication by others?

Yes

Reviewer Expertise:

Regulation of gene expression, TF-mediated DNA demethylation, Cell reprogramming

I confirm that I have read this submission and believe that I have an appropriate level of expertise to confirm that it is of an acceptable scientific standard, however I have significant reservations, as outlined above.

# Response

**Published:** 2021-03-02

**Authors:** Naoya Yahagi

**Affiliations:** Nutrigenomics Research Group, Faculty of Medicine, University of Tsukuba, Japan

*1. Identification of upstream TF(s) should be objective. Test cases shown in Figure 2A, B and C should be statistically evaluated. HIF-1a,2a and SREBP-1a,-2 may be evident, however I am not sure whether LXRa-mediated luciferase activity is significantly high or not.*

 We thank the reviewer for the valuable advice. Because the data have been obtained for the screening purpose, each pool has just single value, so robust statistical analyses to show significance are a little difficult to perform. However, as an empirical method, 2SD (standard deviation) criteria is often used. For Figure 2A (Vegfa-luc), only the pools #5 and #29 (black bars; which means that they actually contain positive clones) are above the 2SD line. For Figure 2B (Fasn-luc), the pools #30 and #60 (black bar) are above the 2SD line, but the pool #30 was revealed to contain no positive clones after the 2nd screening of each clone in the pool. For Figure 2C (Srebf1c-luc), the pools #5, #50, #76 (black bar) and #78 are above the 2SD line, but only the pool #76 was revealed to have a positive clone (LXRα) after the 2nd screening. Our actual strategy was to pick up the top 5% (or the bottom 5% for the search of suppressors as shown in Figure D) for the 2nd screening. As a result, both approaches (the 2SD criterion and the top 5% criterion) are proved to work sufficiently as a screening method.

 Based on this discussion, we added the following paragraph to the revised manuscript:

 From the statistical point of view, we picked up the top (or the bottom for D) 5% pools for the 2nd screening, and as a result, this criterion was similar to the 2SD (standard deviation) criterion in these cases.

*2. In figure 2D, authors selected 4 pools. I think this selection should also be objective by using statistical evaluation. If authors statistically selected pools for further one by one test, the pool with KLF4 has been selected?*

 Thank you for the question. We selected the top or bottom 5% pools for the 2nd screening, and only the pool #28 containing KLF4 was proved to be a true hit after the 2nd screening.

*3. One of the most important value in this work must be TF expression plasmids that authors have systematically constructed. I think this resource should be available to scientific community.*

 Thank you for the comment. We are planning to talk with DNAFORM about how to share this expression library in the near future.

*4. TFs regulate downstream genes by associating with promoters or enhancers. It is worth if the screen system works for TFs associating with enhancers.*

 We thank the reviewer for the valuable comment. Yes, we think that our method is also effective for enhancers to be matched with TFs, as shown in the case of TCF7L2 gene (Piao X, et al, FEBS Lett, 2018;592(3):422-433 (ref. 9)). According to the reviewer’s advice, we added the following sentence to the revised manuscript:

 It is noteworthy that the TFEL scan method is also applicable to enhancers as well as promoters as shown in the case of *TCF7L2* gene.

# Peer review: Reviewer response for version 1

**DOI:** 10.5256/f1000research.30430.r78980

**Published:** 2021-02-09

**Authors:** Kazuhiko Igarashi

**Affiliations:** 1 Department of Biochemistry, Tohoku University Graduate School of Medicine, Sendai, Japan

This is a very important resource paper for the fields of gene regulation, metabolism, differentiation and stress response. Overall, the manuscript is well written and the results are clearly presented. There are some issues readers may like to know better.

1. Human genome is estimated to encode for 1639 transcription factors (Lambert SA et al, Cell 172, 650-, 2018 ^1^). How much of the mouse transcription factors in the expression library is overlapping with the human transcription factors. A brief discussion will be helpful.
2. Many of the transcription factors characterised thus far are responsive to stresses or ligands. The results in this manuscript show that such transcription factors can be identified by overexpression. A brief discussion is needed.
3. How will this expression library be shared?
4. The description in the text of experiment in Fig. 2D may be improved. Did authors try to identify factors that restrict/inhibit LXR? Why was this important for the regulation of Srebf1c promoter?

Is the work clearly and accurately presented and does it cite the current literature?

Yes

If applicable, is the statistical analysis and its interpretation appropriate?

Yes

Are all the source data underlying the results available to ensure full reproducibility?

Yes

Is the study design appropriate and is the work technically sound?

Yes

Are the conclusions drawn adequately supported by the results?

Yes

Are sufficient details of methods and analysis provided to allow replication by others?

Yes

Reviewer Expertise:

gene regulation by transcription factors, their functions in cell differentiation and diseases.

I confirm that I have read this submission and believe that I have an appropriate level of expertise to confirm that it is of an acceptable scientific standard.

## References

[1] : The Human Transcription Factors. *Cell*.2018;172(4) : 10.1016/j.cell.2018.01.029 650-665 10.1016/j.cell.2018.01.029 29425488PMC12908702

# Response

**Published:** 2021-03-02

**Authors:** Naoya Yahagi

**Affiliations:** Nutrigenomics Research Group, Faculty of Medicine, University of Tsukuba, Japan

*1. Human genome is estimated to encode for 1639 transcription factors (Lambert SA et al, Cell 172, 650-, 20181). How much of the mouse transcription factors in the expression library is overlapping with the human transcription factors. A brief discussion will be helpful.*

 We thank the reviewer for the valuable advice. According to this suggestion, we checked the overlapping among the transcription factor databases such as RIKEN TFdb (the original database that the TFEL is based on; Kanamori M et al, Biochem Biophys Res Commun. 2004;322(3):787-793 (ref.7)), Human TF (Lambert SA et al, Cell, 2018;172(4):650-665.), and Animal TFDB 3.0 (Hu H et al, Nucleic Acids Research, 2019;47(D1):D33-D38.), using a gene symbol converter available at http://biodb.jp/. The results were as follows: the overlap between RIKEN TFdb and Human TF was 889 genes, app 65% of Human TF and 53% of RIKEN TFdb, and the overlap between RIKEN TFdb and Animal TFDB 3.0 was 958 genes, app 59% of Animal TFDB 3.0 and 57% of RIKEN TFdb. The differences were a little larger than expected and even when we compared the same Animal TFDB human and mouse databases, the overlap was limited to 1,340 genes, which were app. 80% of the 1665 human TF genes in the database. Therefore, app. 300 TF genes (20%) seemed to be different between human and mouse. Thanks to the reviewer’s comment, we could better understand the differences among TF databases, but because this content is beyond the scope of this article, we will keep it within the Comments. Also, we realized during these series of gene symbol checking that KLF 9, 10, 11 had been included in the TFEL library, and corrected the description in the discussion.

*2. Many of the transcription factors characterized thus far are responsive to stresses or ligands. The results in this manuscript show that such transcription factors can be identified by overexpression. A brief discussion is needed.*

 We thank the reviewer for the valuable comment. According to the reviewer’s advice, we added a new paragraph of discussion to the revised manuscript like this:

 Many of the transcription factors characterized thus far are responsive to stresses or ligands. The results of the case of LXR shown here demonstrate that such transcription factors can also be identified by overexpression. However, at the same time, we have experienced that some transcription factors activated in a ligand-dependent manner, for example, glucocorticoid receptor (GR), cannot be identified by mere overexpression without ligands (data not shown). Therefore, it may depend on transcription factors whether they can be detected without ligands/additional activators or not.

*3. How will this expression library be shared?*

 Thank you for the comment. We are planning to talk with DNAFORM about how to share this expression library in the near future.

*4. The description in the text of experiment in Fig. 2D may be improved. Did authors try to identify factors that restrict/inhibit LXR? Why was this important for the regulation of Srebf1c promoter?*

 Thank you for the question. Actually, there is a long story behind this. To make the long story short, we had found an important element shown to exert a suppressive effect on the SREBP-1c promoter in a fasting state (Takeuchi Y, et al, Cell Rep, 2016;16(9):2373-2386 (ref. 6)). According to the reviewer’s advice, we added this explanation to the revised manuscript.
